# Quantitative Analysis of Motor Status in Parkinson's Disease Using Wearable Devices: From Methodological Considerations to Problems in Clinical Applications

**DOI:** 10.1155/2017/6139716

**Published:** 2017-05-18

**Authors:** Masahiko Suzuki, Hiroshi Mitoma, Mitsuru Yoneyama

**Affiliations:** ^1^Department of Neurology, Katsushika Medical Center, Jikei University School of Medicine, Tokyo, Japan; ^2^Medical Education Promotion Center, Tokyo Medical University, Tokyo, Japan; ^3^MCHC R&D Synergy Center, Inc., Yokohama, Japan

## Abstract

Long-term and objective monitoring is necessary for full assessment of the condition of patients with Parkinson's disease (PD). Recent advances in biotechnology have seen the development of various types of wearable (body-worn) sensor systems. By using accelerometers and gyroscopes, these devices can quantify motor abnormalities, including decreased activity and gait disturbances, as well as nonmotor signs, such as sleep disturbances and autonomic dysfunctions in PD. This review discusses methodological problems inherent in wearable devices. Until now, analysis of the mean values of motion-induced signals on a particular day has been widely applied in the clinical management of PD patients. On the other hand, the reliability of these devices to detect various events, such as freezing of gait and dyskinesia, has been less than satisfactory. Quantification of disease-specific changes rather than nonspecific changes is necessary.

## 1. Introduction: Why Are Wearable Devices Clinically Necessary?

Parkinson's disease (PD) is characterized by poverty of movements (akinesia) and smallness and slowness of executed movements [1–4]. While dopamine replacement therapy can improve these motor abnormalities, patients with PD suffer motor fluctuations at advanced stage [1, 2]. Under these conditions, routine clinical examinations do not provide sufficient clinical information for proper management of these patients, since they can only estimate one point of the condition. Unfortunately, patients cannot visit the hospital at the time of worst motor deficits. In addition to these problems, freezing of gait is hardly observed in the examination room due to increased attention [1–4]. Thus, medical interviews and physical examinations in the clinics often do not provide the full picture of the condition, as they do not include the events at homes. In other words, without information about the condition outside the hospital/clinic, treatment will be less than ideal.

Physicians have been well aware of this aspect of clinical management. To overcome this problem, diaries have been used for proper estimation of Parkinsonian condition throughout the 24 hours [3, 4]. However, such recording is subjective. Patients with cognitive and attention impairments might also neglect fluctuations. Thus, there are still wide gaps in capturing Parkinsonian symptoms between the physicians and patients. For detailed capture of Parkinsonian symptoms and planning a comprehensive treatment strategy, continuous (long-term) and objective recording on Parkinsonian symptoms is necessary.

Recent progress in digital and biotechnology has encouraged the development of many types of wearable sensor systems that can monitor physical activity throughout the 24 hour [5]. Maetzler et al. [5] proposed the following clinical signs as targets for wearable devices; (1) motor disabilities, including axial disability (gait and transfer deficits, freezing of gait, imbalance, and frequencies of falling), bradykinesia in the distal limbs, dyskinesia, resting tremor, dysarthria, and secondary low activities, and (2) nonmotor abnormalities, which include sleep disturbances and autonomic dysfunction [5]. Although many kinds of accelerometers and gyroscopes-mounted sensors have been developed [5], the methodological problems underlying various sensors have not been systematically discussed in detail. For proper and precise interpretation of the results obtained from various sensors, clinicians should understand the principles behind the recordings of various items and the methods used for recording and analysis.

The aim of this article is to provide a review of the methodological problems inherent in each sensor and how such problems affect the clinical applications of the recording devices. Especially, we discuss the problems that are still unsolved for clinical application of each wearable device currently used to quantify daily activities of PD patients.

## 2. Methodological Considerations

Wearable sensor systems have wide applications in a variety of fields, not only in medical science but also in entertainment, fitness, and sports [6]. They are light-weight, usually designed in the form of small devices that can be fixed on the body by bands or enclosed in everyday items, for example, watches, phones, rings, glasses, and clothing [6–9], allowing continuous and unobtrusive monitoring in real-world real-time settings. Basically, a wearable device contains at least one sensor, a signal processor, and a display. Raw signals collected by the sensor are processed, and the results are shown on the display, by which the users can track their physiological data in real time [6]. Some devices are equipped with wireless communication, allowing the data to be transmitted to remote sites (PC, mobile phone, and base-station) where more extensive analysis and display may be conducted. The maximum duration of measurement is determined by the minimum between battery life and the memory capacity of the device. The choice of the sensor to be used will depend on the purpose of monitoring [10]. Examples well used for the detection of human movements are inertial sensors such as accelerometers and gyroscopes.

### 2.1. Accelerometers and Gyroscopes

#### 2.1.1. Accelerometers

Accelerometers detect acceleration induced by body motion. The basic mechanism of measurement of acceleration is explained by a mass-spring system [11–17]; a mass is displaced when acceleration is applied, generating a force in a spring connected to the mass. Then, the acceleration can be obtained by a combination of Hooke's law and Newton's second law [14]. There are several classes of accelerometers currently available depending on the method of signal transduction; the most common are piezoresistive, piezoelectric, and differential capacitive accelerometers [12–17]. Piezoelectric types are only sensitive to dynamic acceleration, while piezoresistive and capacitive accelerometers can respond to both dynamic and static acceleration (e.g., gravity). Such DC-responsive nature is helpful in determining the posture of the subject at rest, since the inclination of the sensor (i.e., the body segment onto which the sensor is attached) relative to the vertical (gravity) vector can be directly calculated from the sensor output [12, 13]. This feature has been exploited in the accelerometry-based identification of basic daily activities (sitting, standing, lying, walking, etc.) [13, 18, 19], sedentary behavior [20], and the occurrence of falls [9, 12]. It is reported that a single waist-mounted accelerometer can distinguish between activity and rest and recognize basic postures (sitting, standing, and lying) and transitions between them [21]. However, accelerometry has certain drawbacks when applied alone for the detection of dynamic events such as gait.

(1) Accelerometers cannot measure rotation around the vertical direction, making it a difficult task to recognize, for example, left and right turns during walking.

(2) In most cases, acceleration along a sensing axis will contain a variable, spurious contribution from gravity induced by the deviation of the axis from the global horizontal. This component cannot be separated from a pure inertial component based on the acceleration output, producing important errors in the process of motion analysis [14, 17].

#### 2.1.2. Gyroscopes

Gyroscopes detect angular velocity of a rotating body by measuring the Coriolis force generated in a rotating reference frame [16, 17]. They can be used alone for human motion capture. For example, gyroscopes attached to the lower limbs provide useful information on gait patterns, such as segment inclination, cadence, step length, and walking velocity [22]. The major drawback of gyroscopes is that they demand high power consumption, which limits the duration of monitoring [23]. Gyroscopes are often concurrently used with accelerometers in order to compensate the above-described limitations of accelerometry and to properly assess dynamic activities. Accelerometers and gyroscopes (and sometimes magnetometers) packaged together constitute an electronic device called inertial measurement unit (IMU), which is one of the most widely employed types of wearable motion sensors.

### 2.2. Physical Activity Monitoring

Physical activity is defined as “any bodily movement produced by skeletal muscles that requires energy expenditure” [24], offering an attractive target for the application of acceleration sensors. In order to capture basic human activities, accelerometers should cover an amplitude range of −12 to 12 g and a frequency range of 0 to 20 Hz [11] (although frequencies up to 60 Hz may be needed to detect foot acceleration at heel strike [12, 25]). The first attempt to objectively measure the level of physical activity by accelerometry dates back to the 1980s [26]. Since then, the accelerometry-based physical activity research has greatly expanded [27], and accelerometers have been considered as the most promising tools for the assessment of physical activity under free-living conditions [11, 28]. Physical activity monitors have been commercially available in the last two decades, for example, ActiGraph (ActiGraph LLC) and StepWatch™ (modus health llc) [10]. Standard output measures from these devices are activity count (which may be arbitrary, developer-specific units [25, 26]) and/or more universal quantities, such as step count and energy expenditure. The ActiGraph and StepWatch are the most commonly used accelerometers for step counting with an accuracy of over 90% [29]. The validity of estimating energy expenditure by accelerometry relies on experimental evidence accumulated over the years that accelerometer output (e.g., the integral of the absolute vertical acceleration at the waist [25]) correlates closely with energy expenditure (or oxygen consumption) [11, 25, 27]. The choice of the model describing this relationship determines the accuracy of estimation. Prediction methods are still under development to achieve better accuracy, from early models using simple linear regressions to more sophisticated approaches based on current advances in technologies such as machine learning and large data computing [26, 30].

Systematic reviews have been published on recent studies of accelerometry-based physical activity monitoring, with a special focus on analytical techniques [31], and long-term monitoring (≥24 h) of healthy elderly [32] and patients with neurological disorders [33].

### 2.3. Gait Analysis

Wearable sensors are widely used for human gait analysis and explore a variety of gait characteristics [16, 34]. The first step in gait analysis is to identify the subject's foot-strike events, which enables straightforward computation of basic gait parameters including the number of steps, step or stride interval (or cadence), gait variability, and asymmetry. Accurate step detection is a precondition for more in-depth gait analysis. It is possible to detect gait steps from a single trunk accelerometer by using such algorithm as template-matching, Pan-Tompkins, Dual-axial, and Wolf method [35, 36]. Recently, more powerful algorithms have been proposed that can extract the initial contact (heel strike) and final contact (toe-off) gait events from a single waist-mounted accelerometer [37, 38] or IMU [39], resulting in proper assessment of fundamental gait phases, that is, stance, swing, and double support. Moreover, by adopting shoe-mounted accelerometry, two more gait events (heel-off and toe strike in addition to heel strike and toe-off) can be precisely identified [40]. Gait variability and asymmetry thus obtained by accelerometers show poor agreement with a laboratory reference (instrumented walkway). Interestingly, this seems to be due to a higher sensitivity of accelerometry that can continuously track whole body motion during walking [38].

The next important parameters of human gait are walking speed, step length, and walking distance. These parameters are readily obtained if we can correctly track the location of a walking subject. In principle, the absolute speed and position of a moving body can be calculated by integrating translational acceleration from motion sensors once and twice over time, respectively. Unfortunately, this is impossible in actual practice, since the integration will amplify even tiny errors in acceleration due to (1) bias drift and noise and (2) spurious contribution from gravity [41], resulting in significant baseline drifts that grow rapidly with time. Such drifts may be eliminated when the integration is combined with high-pass filtering of the signal, but the resulting value is not an absolute position or speed but a “relative” displacement or speed. While it is difficult to determine step length and walking speed directly from acceleration data, several mathematical models have been designed to predict these parameters.

(1) The relative vertical displacement of the trunk while walking is calculated by double integration of the vertical acceleration. This is converted into forward displacement (step length) with the help of an inverted pendulum model of walking [42, 43].

(2) A set of features is extracted from trunk-mounted three-dimensional (3D) accelerometer signals, which are used to compute walking distance or speed using machine learning algorithms [44].

(3) Step length is estimated from step frequency (cadence) [42] based on the linear relationship between the two parameters for normal gait [45].

By its nature, the application of method (3) is not restricted to (trunk-mounted) accelerometry; it can be expanded, with a combination of adaptive algorithms, to inertial devices attached on other parts of the body, for example, foot-mounted accelerometers [46], wrist-mounted (e.g., smart-watch) [47], or handheld [48, 49] IMUs. Furthermore, in this case, it is not necessary to detect step events from sensor signals for identifying step frequency; instead, the power spectrum of the signal may provide satisfactory information on step frequency [46, 48].

The errors inherent in the integration of acceleration can be significantly reduced when foot-worn IMUs are adopted for position tracking [50].

(1) In order to eliminate the spurious component due to gravity, the sensor signals in the device frame are transformed to the global reference frame by using a coordinate transformation matrix. The matrix, usually in the quaternion representation, can be obtained by integrating the gyroscope output [51].

(2) The velocities along the three axes are computed by integrating the acceleration in the global frame, which include errors due to bias drift and noise. While walking, the foot is in contact with the ground for a short period of time, during which all dynamic acceleration and velocities of the foot-worn sensor should be zero. Then, the calculated velocities can be reset to zero at the time of every foot-contact event, meaning that the integration errors do not accumulate over time. This technique is called “zero velocity update” [52].

The 3D-foot kinematics can be fully reproduced in this way, offering an accurate estimate of not only step length and walking speed but also other spatiotemporal gait parameters, such as foot clearance and turning angle [53]. The IMU-based kinematic analysis constitutes the basis for personal dead-reckoning [50, 54], a process of tracking the route taken by a walking individual without GPS. Moreover, it can be extended, together with a suitable biomechanical model of the human body, to the case where multiple sensors are located on different segments of the body in addition to feet. For example, by analyzing the signals from seven IMUs placed on the lower limbs (feet, tibias, thighs, and pelvis), the entire leg kinematics has been accurately reconstructed in a hierarchical manner, from feet to tibias, to thighs, and to hips [55]. Furthermore, Xsens MVN, a full-body motion tracking suit, has been commercialized, which consists of 17 IMUs and can capture any body movement including walking and running [56].

Wearable sensors have made it possible to perform a thorough comparison between clinical laboratory and free-living gait analysis. It was found that free-living gait exhibits lower cadence and higher variability than laboratory-assessed gait [57, 58]. This finding suggests that laboratory evaluation may reflect the subject's optimal performance, not his/her typical performance [57]. In addition, long-term free-living data have clarified the importance of “bout” in gait analysis, which is defined as the time period spent in continuous walking. Del Din and coworkers [58] recommended that gait characteristics should be obtained over longer bouts (e.g., a minimum of 10 sec) in order to discriminate between normal and PD gait. Weiss et al. [59, 60] extracted bouts with duration of ≥1 min from 3-day accelerometer recordings and calculated several gait measures from these bouts.

### 2.4. Single versus Multiple Sensors

The number of sensors to be used depends on the trade-offs between several factors, for example, cost, usability, subject compliance, and (mostly) the purpose of monitoring [12]. No doubt multiple sensors provide more information on body movements, facilitating a better understanding of human activities. A critical challenge along this line is the Wireless Body Area Network, which is a network of miniaturized, wireless sensors on or around a human body to monitor body function and environment of the wearer and serves as a core technology in ubiquitous healthcare systems [61–63]. The complexity of wearing many sensors could be alleviated by integrating all sensing items into clothing [56, 64].

Nevertheless, it is desirable to adopt the minimum number of sensor units, considering ease of use and cost effectiveness. Reducing the number of sensors is a wise choice particularly when the quality of information is not affected; that is, the same set of outcome measures can be obtained from fewer sensors with the help of sophisticated analytical algorithms and mathematical modeling [65]. The most extreme approach is to use only one sensor attached at a single location on the body [12]. A tutorial is available on how to conduct practical gait assessment by a single, low-cost, open source wearable sensor [66]. Moreover, the single sensor-based motion analysis has been gaining popularity recently due to the availability of affordable, off-the-shelf smart devices, such as the smartphones and smartwatches with built-in inertial sensors [67, 68]. The smart devices are ideal for unobtrusive monitoring, because they are now becoming everyday companions for many people and do not interfere with the normal daily activities of the user, compared to stand-alone inertial sensors. Current research on applying such consumer devices to motion analysis is centered on two important issues. One is to validate the performance of the device against established (gold standard) methods [68–70]. In this case, especially in gait analysis, trunk-mounted configuration is selected, and the results seem very promising. Another issue is to cope with the phone context problem [67, 71]. Namely, the position of the smart phones constantly changes depending on the context; they are placed in the pocket, in the bag, or handheld for phoning, texting, and so forth [49, 72]. Any analytical algorithms developed on the premise of a fixed sensor position will fail to work in such a situation. Therefore, robust, adaptive algorithms need to be designed that can recognize automatically different device poses and human activities.

## 3. Clinical Applications

### 3.1. Technologies for Monitoring PD Motor Status

Various wearable technologies have been proposed for the assessment of various domains of the motor status in PD, for example, tremor, freezing of gait, falls, postural instability, bradykinesia, and dyskinesia [5, 23, 73–76].

Ullah et al. [61] conducted a systematic review of the current literature on technology-based devices used for evaluation of PD. They classified the following wearable devices as “recommended”: Mobility Lab™ [77, 78], Physilog® [79], StepWatch 3, TriTrac RT3, DynaPort [80, 81], and AX3 [82]. The Personal Kinetograph (PKG) has also been widely used in the clinical management of PD patients. These devices are designed to record changes in overall movements (voluntary limb and trunk movements-induced signals) and analysis of gait disorders.  Table 1 summarizes the main features of the above devices.

Recent advances in machine learning have helped to uncover important clinical information hidden in the sensor data. For instance, Klucken et al. [83] proposed Embedded Gait Analysis using Intelligent Technology (eGaIT) to monitor motor signs of gait impairment in PD. The eGaIT system extracts a total of 694 gait features from signals recorded with shoe-mounted IMUs, onto which pattern recognition algorithms are applied to classify different H&Y stages or different levels of motor impairment (UPDRS-III).

Monitoring the effects of treatment is another focus of recent studies. Furthermore, wearable technologies have been employed to provide PD patients with real-time training on their motor performance in the home environment [84]. In addition, while inertial sensing systems are very powerful tools for monitoring motor disturbances, they can also be applied to recognize nonmotor disabilities including sleep disorders and autonomic dysfunction [5]. Moreover, it may be possible to capture, though indirectly, cognitive aspects based on these systems because human locomotion, which can be directly assessed by inertial sensors, is tightly linked to cognition [75].

### 3.2. Clinical Studies

As discussed in a previous section, numerous studies have successfully and accurately recorded well-known motor abnormalities in PD using wearable devices. Maetzler et al. [5] reviewed the validity of clinical applications of these pilot studies. Their review can be summarized as follows: (1) poverty of overall movements and smallness/slowness of executed movements can be estimated as decrease in motion-induced acceleration. (2) Parkinsonian gait features can be characterized by decrease in stride length, slowness in cadence, and decrease in walking speed. (3) Freezing of gait can be captured as an abrupt change in freezing-induced acceleration signals, which are different from normal gait-induced signals.

These estimations are reasonable, since most studies quantify the features that can be identified by physical examination. On the other hand, Weiss and colleagues have selected features that can be identified exclusively using accelerometer-mounted wearable devices [59, 60, 85–87]. They calculated the gait cycle time and the step-to-step variability in PD patients [59, 60, 85–87]. They reported higher step-to step variability in fallers than nonfallers [59, 86] and in freezers than nonfreezers [60].

### 3.3. Open Problems

As discussed in a previous chapter, movement-induced acceleration or gyroscopes have been used to monitor the motor features of PD. Several studies have quantified various abnormalities recorded during unrestrained daily activities of patients with PD. These studies suggest the existence of two major problems with signal validity.

The first problem is the location of sensors. The sensor may be placed on the body part from which the target motion can be most effectively measured. However, there is as yet no consensus among researchers regarding the best anatomical area for sensor placement in PD patients [23, 74]. Let us take freezing of gait as an example, which is associated with rapid trembling in the legs. Earlier research attempted to detect this anomalous movement by inertial sensors attached on the lower limbs (i.e., shank, thigh, and foot) [88]. In recent years, research has focused on other areas for easier mounting of the sensor, such as waist [89, 90] and wrist [91]. In this regard, a recent survey on user preferences for placement of such devices on the body found that the wrist, arm, and waist are much preferred than lower limbs among the patients (in this case, patients with osteoarthritis) [92].

The second problem is reliability of the detected events. For example, a device that continuously monitors gait-induced acceleration accurately estimates the mean values of amplitude (force) and cycle (rhythm) of gait acceleration on a particular day [93, 94]. By using these mean values, the bradykinetic features of daily walking can be successfully quantified [93, 94]. In contrast, in spite of exhaustive efforts to detect freezing of gait, the sensitivity and specificity of such device remain to be improved [5]. Although some algorithms have been proposed to improve the detection of abrupt changes in frequencies or attenuation in amplitude, both of which are characteristic features of freezing, the sensitivity and specificity are less than satisfactory [5]. Similarly, it is difficult to capture dyskinesia. The abrupt increase in activities suggests occurrence of dyskinesia [93]. However, patients voluntarily move their limbs with abruptly increased acceleration in their daily lives. The kinematic feature of abrupt voluntary limb movement closely resembles that of dyskinesia [93, 94]. Freezing associated with wearing off and drug-induced dyskinesia is an important problem in the management of patients with PD. No doubt further developments in this field are needed.

Finally, to identify the exact pathophysiological changes in PD, wearable devices should quantify disease-specific changes rather than nonspecific changes. Previous studies showed that quantification of PD-specific deficits in production of propelling forces or setting of step rhythm can exactly capture the severity of abnormality in PD patients, compared with the capture of nonspecific changes in gait parameters, such as decrease in stride, cadence, or velocity [93, 94]. Algorithms that can detect PD-specific changes in motor deficits are desirable.

In conclusion, many efforts to detect activities or gaits have been accomplished using accelerometer- or gyroscopes-mounted wearable devices. Technological advances in the construction of these devices have opened the door to new era in which motor status can be assessed in daily living. However, more reliable sensitivity and quantification of PD-specific pathophysiological changes should increase the clinical value of wearable devices and consequently enhance our understanding of the clinical condition and help in the selection of effective therapeutic strategies. Further studies are needed to conduct the same studies in large number of control patients.

## Figures and Tables

**Table 1:** Features of currently available wearable devices.

	Type of sensor(s)	Features
*Devices that mainly monitor overall activities*		
TricTrac RT3	Accelerometer- and gyroscope-mounted sensor	Recording of overall activities in kinematic or kcal mode

*Devices that mainly monitor gait disorders*		
StepWatch	Specific ankle-worn microprocessor-based step counter	Long-term gait analysis (50 days at 60 sec resolution)
DynaPort	Accelerometer-mounted sensor attached on the trunk	Long-term gait analysis (14 days)

*Devices that monitor changes in overall activities and gait disorders*		
Mobility Lab	Accelerometer- and gyroscope-mounted sensor attached on limbs and trunk	Recording of overall activities and analysis of gait and posture
Physilog	Accelerometer-, gyroscope-, and barometric pressure-mounted sensors attached on limbs and trunk	Recording of overall activities and analysis of gait and running
AX3	Accelerometer-mounted sensor attached on limbs or trunk	Recording of overall activities and analysis of gait and running
